# Cortical structural network characteristics in non-cognitive impairment end-stage renal disease

**DOI:** 10.3389/fnins.2024.1467791

**Published:** 2024-11-13

**Authors:** Yimin Wang, Shihua Chen, Peng Zhang, Zixuan Zhai, Zheng Chen, Zhiming Li

**Affiliations:** ^1^Department of Radiology, The Second Affiliated Hospital of Guangzhou Medical University, Guangzhou Medical University, Guangzhou, China; ^2^Department of Organ Transplantation, The Second Affiliated Hospital of Guangzhou Medical University, Guangzhou, China; ^3^Qinghai Cardio-Cerebrovascular Specialty Hospital, Qinghai High Altitude Medical Research Institute, Xining, China

**Keywords:** structural covariance network, end-stage renal disease, neuroimaging, graph theory, cortical volume

## Abstract

**Objective:**

Explore alterations in topological features of gray matter volume (GMV) and structural networks in non-cognitive impairment end-stage renal disease (Non-CI ESRD).

**Materials and methods:**

Utilizing graph theory, we collected structural magnetic resonance imaging (sMRI) data from 38 Non-CI ESRD patients and 50 normal controls (NC). We compared, and extracted the GMV across subject groups, constructed corresponding structural covariance networks (SCNs), and investigated the alterations in SCNs feature parameters between groups.

**Results:**

In Non-CI ESRD patients, The GMV were reduced in several brain regions, predominantly on the left side (*p* < 0.05, FWE correction). The small-world network characteristics of the patient group’s brain networks showed a tendency toward regular. In a few densities, global network parameters, transitivity, (*p* < 0.05) was significantly increased in the ESRD group. Regional network measurements revealed inconsistent changes in regional efficiency across different brain areas. In the analysis of network hubs, the right temporal pole is likely a compensatory hub for Non-CI ESRD patients. The SCNs in Non-CI ESRD patients demonstrated reduced topological stability against targeted attacks.

**Conclusion:**

This study reveals that patients with renal failure exhibited subtle changes in brain network characteristics even before a decline in cognitive scores. These changes involve compensatory activation in certain brain regions, which enhances network transitivity to maintain the efficiency of whole-brain network information integration without significant loss. Additionally, the SCNs characteristics can serve as a neuroanatomical marker for brain alterations in Non-CI ESRD patients, offering new insights into the mechanisms of early brain injury in ESRD patients.

## 1 Introduction

Chronic kidney disease (CKD) is a pathological state characterized by persistent and progressive deterioration of renal function, manifested by albuminuria or a glomerular filtration rate (GFR) of less than 60 mL/min/1.73 m^2^ for at least 3 months. A reduction in GFR to 30 or 15 mL/min/1.73 m^2^ indicates advanced or ESRD (Kalantar-Zadeh et al., 2021). In 2017, the number of patients across all stages of CKD reached 700 million, with 1.2 million deaths attributable to the diagnosis of CKD. It is projected that by 2040, this number will rise to 2.2 million at best and could reach 4 million at worst (GBD Chronic Kidney Disease Collaboration, 2020). Effective preventive and therapeutic measures are crucial to mitigate the rising prevalence rate, given the growing public health concern.

It is widely recognized that CKD is one of the major risk factors for cognitive impairment (CI) (Scheppach et al., 2023). Early-stage manifestations may include cognitive decline in various domains such as orientation, attention, and language (Li et al., 2022). Consequently, there is a focus on researching cerebrovascular and morphological changes in the brains of ESRD patients.

The pathophysiological mechanisms underlying CI in ESRD patients remain unclear (Yue et al., 2021), and there are limited data regarding its etiology and brain morphological manifestations (Scheppach et al., 2023). The multi-dimensional cognitive decline observed in ESRD patients is currently thought to be associated with factors such as metabolite deposition (particularly urotoxin), oxidative stress, cerebrovascular inflammation, and electrolyte and fluid imbalances (Rosner et al., 2021). These factors may exert diffuse effects on brain structures. Vogels et al. (2012) have shown that CKD is likely associated with brain lesions including white matter (WM) lesions, silent cerebral infarction (SCI), and cerebral atrophy (CA). Regarding brain gray matter morphology, ESRD patients exhibit a significant reduction in volume within the limbic system (LS) and the default mode network (DMN) (Zhang et al., 2013). Investigating potential imaging biomarkers in patients with early-stage Non-CI ESRD is of paramount importance for early identification, prevention, and the development of intervention strategies.

Nowadays, the convergence of network and neuroscience has given rise to the field of brain network analysis. This field categorizes different cortical regions into structural or functional networks based on their macroscopic connections or correlated functional activities (Richmond et al., 2016), Presented by structural MRI (sMRI)/diffusion tensor imaging (DTI) and functional MRI (fMRI) respectively. The application of graph-theoretic models to brain imaging techniques for brain network analysis is a current hotspot in brain science research. As a common framework, these models facilitate the comparison of results across different modalities of research, and they only require deriving a few simple metrics to describe the structural or topological properties of brain networks holistically (Rubinov and Sporns, 2010).

Resting-state functional magnetic resonance (Rs-fMRI) studies have indicated that ESRD patients not only show abnormal regional brain functional activity but also exhibit a decrease in both intra and inter regional functional connectivity. For instance, the study by Yue et al. (2021) shows reduced node betweenness in regions such as the DMN and bilateral superior frontal gyrus (SFGmed), likely contributing to the decline in cognitive test performance observed in ESRD patients with CI. Changes in brain regions related to the DMN are most pronounced (Liang et al., 2013; Luo et al., 2016; Ni et al., 2014; Zheng et al., 2014). These alterations in neurofunctional networks highly overlap with regions experiencing a reduction in cortical structural volume.

The research by Seeley et al. (2009) further corroborates that large-scale distributed structural networks tend to converge with intrinsic functional networks. This finding suggests that structural covariance networks can macroscopically reflect changes in functional networks. Such a marker can intuitively and effectively represent the alterations in cognitive brain morphology of ESRD patients, thereby providing robust support for the early identification and management of cognitive deficits associated with the disease.

SCNs represent a methodology that elucidates the organizational patterns of brain networks by assessing the covariance of morphological imaging indices across brain regions within a population. SCNs are constructed through the computation of morphological data correlations between different brain areas in a cohort, thereby highlighting the co-variance of brain morphological traits (Alexander-Bloch et al., 2013). These networks capture the impact of unique environmental factors and chronic disease states on the brain’s network architecture, offering stable metrics indicative of long-term network properties. The application of SCNs has been extensive in the investigation of diverse neurological and psychiatric conditions, including Alzheimer’s disease, multiple sclerosis, dyslexia, and the morphogenesis of the fetal brain (He et al., 2008; Wang et al., 2022; Hosseini et al., 2013; He et al., 2009).

In this study, leveraging MRI and graph-theoretic analysis with cortical volume parameters, we compared the large-scale cortical structural networks between ESRD patients and NC. We hypothesized that ESRD patients might exhibit abnormal changes in cortical volume and speculated the presence of shared or distinct structural covariance network patterns. Building on this hypothesis, we further examined the differences in connectivity of network hubs (highly connected nodes) and in regional network efficiency, focusing on node betweenness and degree separately. Although foundational research exists on Rs-fMRI and white matter structural networks DTI imaging (Yue et al., 2021; Hu et al., 2024; Chou et al., 2019), we offers a rarer exploration into the large-scale cortical volumetric covariance networks in Non-CI ESRD patients compared with NC. This comparison aids in a deeper understanding of the precise and coordinated histomorphic changes in the brain’s structural networks of Non-CI ESRD patients and provides an anatomical foundation for the interconnections among pathological alterations.

## 2 Materials and methods

### 2.1 Participants

Between January 2023 and January 2024, 38 Non-CI ESRD patients (ESRD group) and 50 NC group, demographically matched for age, gender, were enrolled at the Second Affiliated Hospital of Guangzhou Medical University for participation in this study. All participants were fully capable of independently administering the MMSE, MoCA. The inclusion criteria for the ESRD group: (1) clinically diagnosed ESRD patients meeting the Kidney Disease Outcome Quality Initiative’s (KDOQI) 2003 criteria for CKD (Levey et al., 2003); (2) age between 30 and 60 years; (3) MMSE scores ≥27, MoCA scores ≥26 (Folstein et al., 1975; Nasreddine et al., 2005); and (4) right-handed.

The exclusion criteria for ESRD group: (1) patients with unstable conditions or those who had undergone renal transplantation; (2) acute renal failure or acute infectious disorders; (3) organic or functional lesions of brain such as severe traumatic brain injury, cerebral infarction, brain tumour, and cerebral hemorrhage, schizophrenia, etc.; (4) severe heart failure, liver disease, etc.; (5) a history of substance dependence or abuse including drugs, alcohol, or illicit substances; and (6) contraindications for MRI scanning such as the presence of a pacemaker, internal metallic foreign bodies, or claustrophobia.

The study was approved by the Ethics Committee of the Second Affiliated Hospital of Guangzhou Medical University. Prior to the commencement of the research, the trial procedures were thoroughly explained to the participants and their legal guardians, who provided consent through a signed informed consent form.

### 2.2 MRI data acquisition and pre-processing

3D-T_1_WI and associated plain scan sequence images were collected using a Philips Ingenia 3.0 T MRI device (Philips Ingenia Elition, AMS). 3D-T_1_WI scans were performed using a 3D-spoiled gradient echo sequence with acquisition parameters: echo time (TE) =3.5 ms, repetition time (TR) = 7.9 ms, field of view = 250 mm × 199 mm × 170 mm, flip angle = 8 degrees, matrix = 252 × 200, slice thickness = 1 mm, number of slices = 170, interslice gap = 0 mm, and number of excitations = 1, with a scan duration of 4 min 41 s.

The raw 3D-T_1_WI image data underwent voxel-based morphometry (VBM) analysis on the MATLAB R2018a data processing platform.^1^ Preprocessing of all subjects’ 3D-T_1_WI images was conducted using the CAT12 tool. The specific steps included:

1. Image format conversion: Converting the original 3D-T_1_WI MRI data files from .dcm to .nii format.
2. Spatial normalization: Refining the converted .nii format data using the SPM12 and CAT12 toolkits for segmentation, reconstruction, correction, and alignment, registering to the Montreal Neurological Institute (MNI) standard space template. The automated anatomical labeling (AAL) template was used to segment the cortical volume, with the 90 cortical and subcortical regions defined by the AAL template designated as regions of interest (ROIs) for VBM analysis.
3. Image quality control and consistency assessment: Utilizing the CAT12 tool for an extensive quality check of the grey matter volume data to identify and exclude any substandard data.

### 2.3 Construction of structural covariance networks

**Figure 1:**
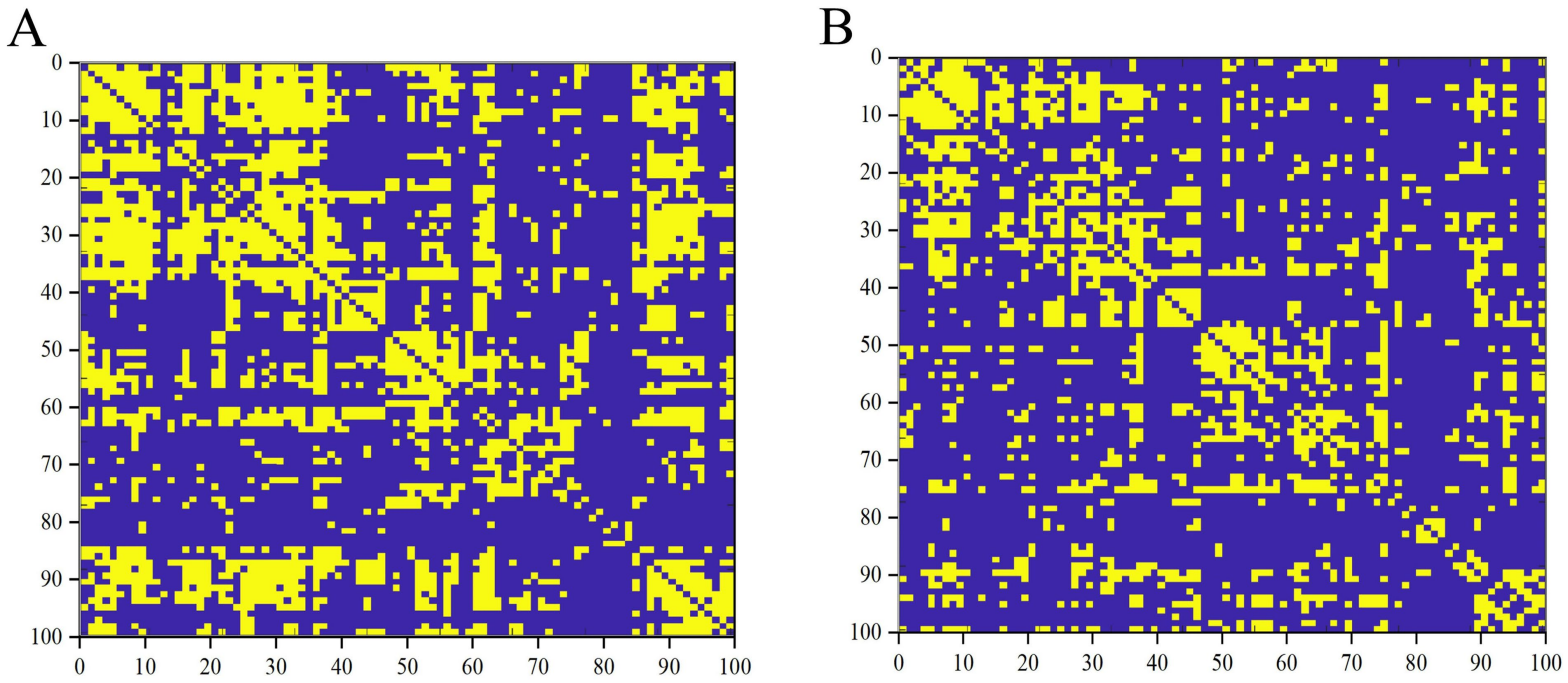
Binary matrices of ESRD group **(A)** and NC group **(B)** over a range of densities (*D* = 0.33–0.50, 0.01).

Using the graph analysis toolbox (GAT), the 90 grey matter volumes of the cerebral cortex and subcortical regions of each subject were extracted with the AAL template and input to GAT to construct SCNs of grey matter volume. The 90 regions of interest in the AAL atlas were defined as nodes, and the edge strength of the SCNs was determined by the Pearson correlation between the grey matter volume values in corresponding brain regions across all subjects, with age, gender, and total intracranial volume (TIV) as covariates. Each entry *r_ij_* was defined as the Pearson correlation coefficient between the grey matter volumes of regions *i* and *j*. The correlation matrix was transformed into a binary matrix, with entries being 1 or 0. The threshold of 0.1 is a standard choice in the field (Yang et al., 2021), confirmed suitable for this study via backpropagation. An entry *a_ij_* is 1 if *r_ij_* exceeds 0.1, otherwise it is 0. Finally, two sets of 90 × 90 binary correlation matrices were output, with the diagonal elements of the constructed matrices also set to 0 (Figure 1). When applying absolute thresholds to threshold the correlation matrices of different groups, variation in node count and degree distribution may occur, affecting network measurements and reducing the interpretability of inter-group comparison results. We therefore set the threshold for each group’s correlation matrix to achieve a binary adjacency matrix with a network density of *D*. The network density *D* is defined as the number of edges in the graph divided by the maximum possible number of edges. Comparing graph measurements requires a minimum density of graph to ensure full connectivity and non-fragmentation. For structural networks, densities exceeding 50% may have questionable biological significance. The network density is calculated as *D* = *E*/[(N*(*N* − 1))/2], where *E* is the number of edges and *N* represents 90 nodes (Hosseini et al., 2012). The minimum density of both networks is calculated to be 0.33, so the lower limit *D*_min_ = 0.33 is defined as the minimum density of the non-fragmented network and the upper limit of the density range *D*_max_ = 0.50 represents the maximum density of the network without exceeding the biological significance. Therefore the density range was taken from 0.33 to 0.50 and verified to be statistically significant and the optimal operating density interval of the network was 0.01, so the density range of the network was determined in increments of 0.01.

### 2.4 Network analysis

#### 2.4.1 Small-world network

The concepts of clustering coefficient (*C*_p_) and characteristic path length (*L*_p_) describing the properties of a small-world network were initially introduced by Watts and Strogatz (1998). *C*_p_ is the average of the clustering coefficients of all nodes in the network and measures the tendency of a node’s neighboring nodes to connect with each other, reflecting the degree of local clustering (Maslov and Sneppen, 2002; Liao et al., 2017). The *L*_p_ of a network represents the average shortest path length between any two nodes. To address issues arising from networks comprising more than one component, Newman and Girvan (2004) and Sporns (2013) proposed measuring *L*_p_ using the harmonic mean distance between pairs of nodes. *L*_p_ (in the form of 1/*L*_p_) quantifies the ability to propagate information in parallel or assess global efficiency (Watts and Strogatz, 1998; Latora and Marchiori, 2001). To evaluate the brain’s topology, these parameters must be compared against the average values of a benchmark random graph (Maslov and Sneppen, 2002; Milo et al., 2002). The small-world metric of the network is obtained from [*C*/*C*_rand_]/[*L*/*L*_rand_], where *C*_rand_ and *L*_rand_ are the average clustering coefficient and characteristic path lengths, respectively, of *m* random networks (Milo et al., 2002). *m* represents the number of null networks used for the standardization of clustering and path lengths, with a default value of 20. *C*_p_ that are significantly higher than random networks (*C*/*C*_rand_, denoted by *γ* in this paper, >1) and *L*_p_ that are comparable to random networks (*L*/*L*_rand_, denoted by *λ* in this paper, ≈1) are considered to satisfy the small-world network property ([*C*/*C*_rand_]/[*L*/*L*_rand_], denoted by *σ* in this paper, i.e., *γ*/*λ* > 1)(Maslov and Sneppen, 2002; Liao et al., 2017).

**Figure 2:**
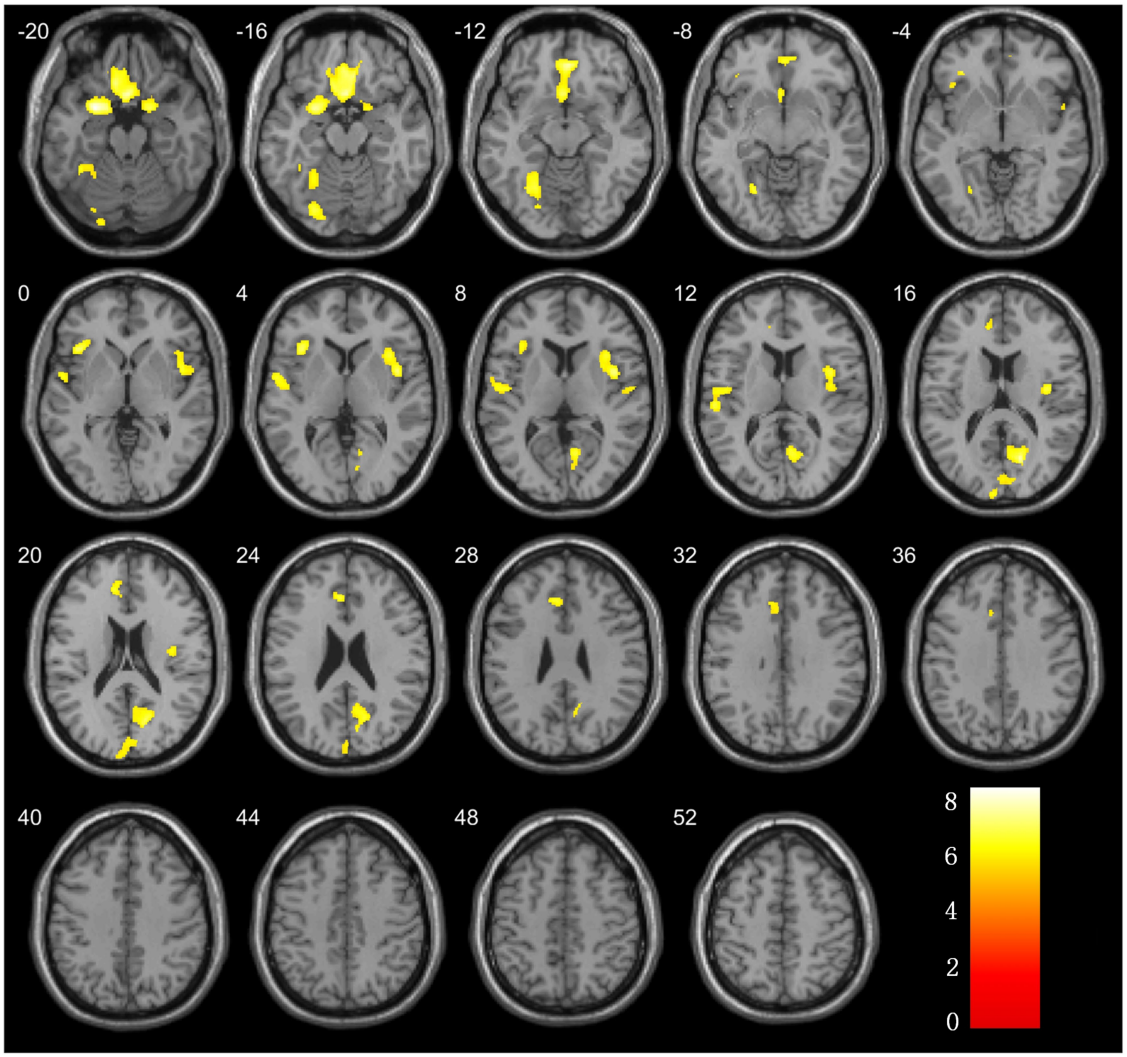
Brain regions with significant differences in grey matter volume comparison between ESRD and NC groups.

#### 2.4.2 Regional network characteristics

The nodal characteristics of regionally structured networks were correlated with changes in RE, including node betweenness centrality and node degree. Analyze the node characteristics, including node betweenness centrality and node degree, of the regionally structured cortical networks. Calculate the normalized regional network measures for each node at a threshold of minimum density *D* = 0.33 to identify between-group differences in these metrics. This approach aims to explore whether there are statistically significant differences between groups regarding regional network metrics. The chosen threshold *D* = 0.33 ensures the inclusion of all regions within the cortical network, effectively reducing the number of false positive connections. By restricting the network in this manner, the correlation strength between regions is optimized, which is considered reasonable for the purposes of this study (Wu et al., 2018).

#### 2.4.3 Network hubs

Network hubs are defined as those nodes with a node betweenness that exceeds the average network node betweenness by more than 2 standard deviations (Hosseini et al., 2012; Yang et al., 2021). They are the most connected regions of the whole-brain network, playing a crucial role in coordinating brain functions through their extensive connections. Network hubs are considered to be important regulators of information flow and, in addition, are key to the network’s resilience to brain injury.

#### 2.4.4 Network robustness

This paper conducts separate analyses for targeted and random attacks. Targeted attacks are a strategy that selectively removes the most critical nodes in the network, ranked by node degree. This strategy models an assault on the most influential elements of the network. In contrast, random attacks indiscriminately remove nodes, chosen completely at random. This simulates scenarios of random failures or attacks (Hosseini et al., 2012). Robustness is the capacity of a system, model, network, or structure to retain its functionality and performance in the face of changes, disturbances, errors, or attacks. In essence, robustness assesses a system’s stability and reliability under adverse conditions (Liao et al., 2017).

### 2.5 Statistical analysis

#### 2.5.1 Demographic and clinical data

To compare the clinical data differences between the ESRD and NC groups, this study utilized SPSS 27.0 for statistical analysis. Age, education level, MoCA, MMSE, scores of both groups underwent normality and homogeneity of variances testing. Metric data conforming to normal distribution characteristics were evaluated for group differences using the two-independent sample *t*-test. Data not conforming to these characteristics were examined using the Mann–Whitney *U* test, a non-parametric alternative. For categorical variables, the chi-square test was performed to assess distribution between the two groups. A *p*-value of less than 0.05 was considered statistically significant.

#### 2.5.2 VBM analysis

In the statistical analysis of VBM data, age, gender, and total brain volume were included as covariates in the analysis to control for potential confounding factors. Two-sample *t*-tests between groups were performed using SPM12 software to compare voxel-wise GMV between the ESRD and HC groups. VBM data processing incorporated family-wise error (FWE) correction with a cluster size threshold of >300 voxels, and differences were considered statistically significant when *p* < 0.05. Finally, the xjView toolbox was utilized to visualize regions of significant difference in brain maps post-VBM analysis.

#### 2.5.3 Analysis of SCNs data

Intergroup comparisons of network metrics were conducted using GAT, focusing on values at *D*_min_ and AUC across the density range from *D*_min_ to *D*_max_. The statistical significance of differences in global and region network metrics was assessed using permutation tests with 1,000 cycles. Results were adjusted for false discovery rate (FDR) at a threshold of *p* < 0.05, two-tailed. A node was deemed a network hubs if its node mediativity was at least 2 standard deviations above the network’s average node betweenness (Hosseini et al., 2012).

## 3 Result

### 3.1 Comparison of demographic and clinical information

A total of 38 cases were included in the ESRD group and 50 in the NC group (Table 1). Differences in gender, age, MoCA and MMSE scores between the two groups were not statistically significant (*p* > 0.05).

**Table 1:** Demographic and clinical characteristics and neuropsychological test scores.

Variables	ESRD group *n* = 38	NC group *n* = 50	Statistic value	*p*
Gender (Female/male)	18F/20M	28F/22M	*χ*^2^ = 0.645	0.422
Years of education (year)	12.47 ± 3.46	13.62 ± 2.79	*χ*^2^ = 7.772	0.169
Age (year)	47.13 ± 9.77	46.82 ± 10.97	*Z* = −0.004	0.997
MoCA	27.21 ± 2.42	28.06 ± 2.01	*Z* = −1.682	0.093
MMSE	28.11 ± 1.18	28.06 ± 1.71	*Z* = −0.298	0.766

The chi-square test was applied to gender and years of education, while the nonparametric rank sum test was applied for age and the rest of the scores. *χ*^2^, chi-square test; *Z*, Wilcoxon rank sum test.

### 3.2 Statistical comparison of cortical volumes

The analysis of the VBM data using two-sample *t*-tests revealed that, compared with the NC group, the ESRD group exhibited decreased GMV in multiple brain regions, with significant reductions in the left temporal pole: superior temporal gyrus, right gyrus rectus, right calcarine fissure and surrounding cortex, bilateral insulae, right parahippocampal gyrus, left superior temporal gyrus, left anterior cingulate and paracingulate gyri, and left fusiform gyrus (FWE-corrected, *p* < 0.05, cluster size>300, *t*-value = 4.96). Specific brain regions, with their MNI spatial coordinates, *p*-values, and associated volume differences, were visualized in three-dimensional images (Table 2 and Figure 2).

**Table 2:** Brain regions with differences in grey matter volume in ESRD group compared to NC group.

Brain region (acronyms)	Voxel (mm^3^)	MNI coordinates			*p*
		*X* coordinate	*Y* coordinate	*Z* coordinate	
TPOsup.L	738	−27	6	−21	<0.001
REC.R	1,884	3	38	−14	<0.001
CAL.R	1,066	16	−69	16	<0.001
INS.R	1,102	42	3	8	<0.001
INS.L	375	−38	22	2	<0.001
FFG.L	943	−28	−62	−14	<0.001
PHG.R	308	20	6	−21	<0.001
STG.L	544	−56	−24	12	<0.001
ACG.L	366	−4	33	26	<0.00

Full names of the corresponding brain regions are provided in Supplementary Table S1.

**Figure 3:**
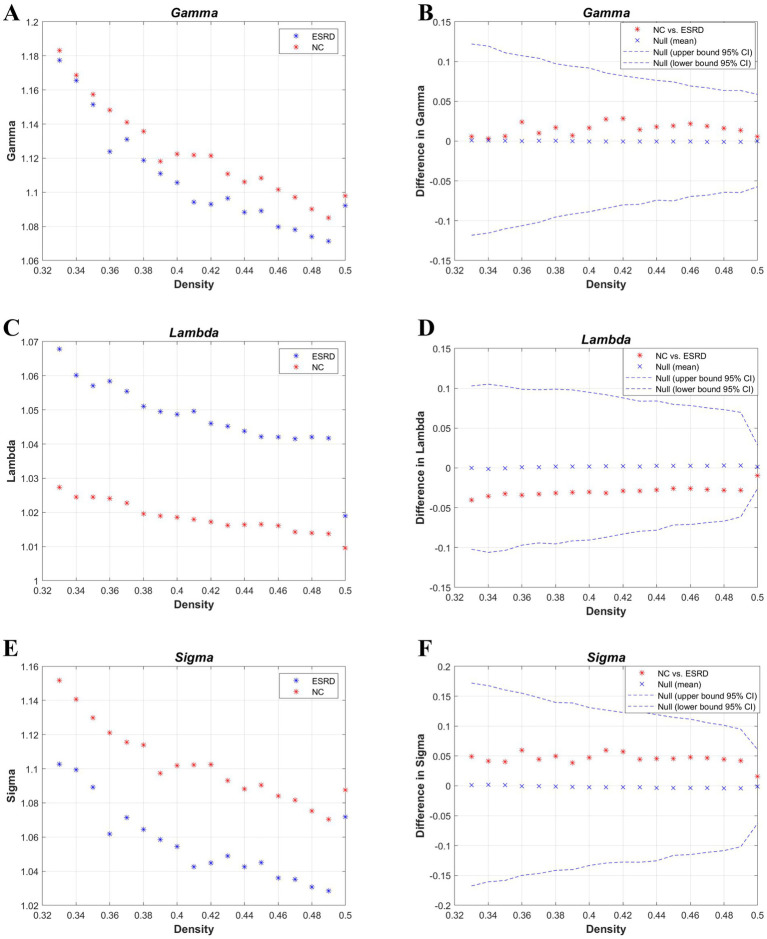
Metrics of small-world properties of the cortical volume SCN for the NC and ESRD groups. [Normalized clustering coefficient (*γ*: gamma) **(A,B)**; normalized path length (*λ*: lambda) **(C,D)**; small-world property (*σ*: sigma) **(E,F)**].

### 3.3 Differences between the global networks

First, binary correlation matrices were output for each group, revealing significant correlations in the brain region structures within both groups (Figure 1).

According to the values of the *γ*, *λ*, and *σ* parameters for both groups (Figure 3), the cortical volume SCNs of both the ESRD group and the NC group exhibited small-world network properties. Although there are no significant differences between the two groups, in the ESRD group, the normalized clustering coefficient (*γ*) and the small-world attribute (*σ*) decrease under a majority of densities, while the normalized characteristic path length (*λ*) increases.

**Figure 4:**
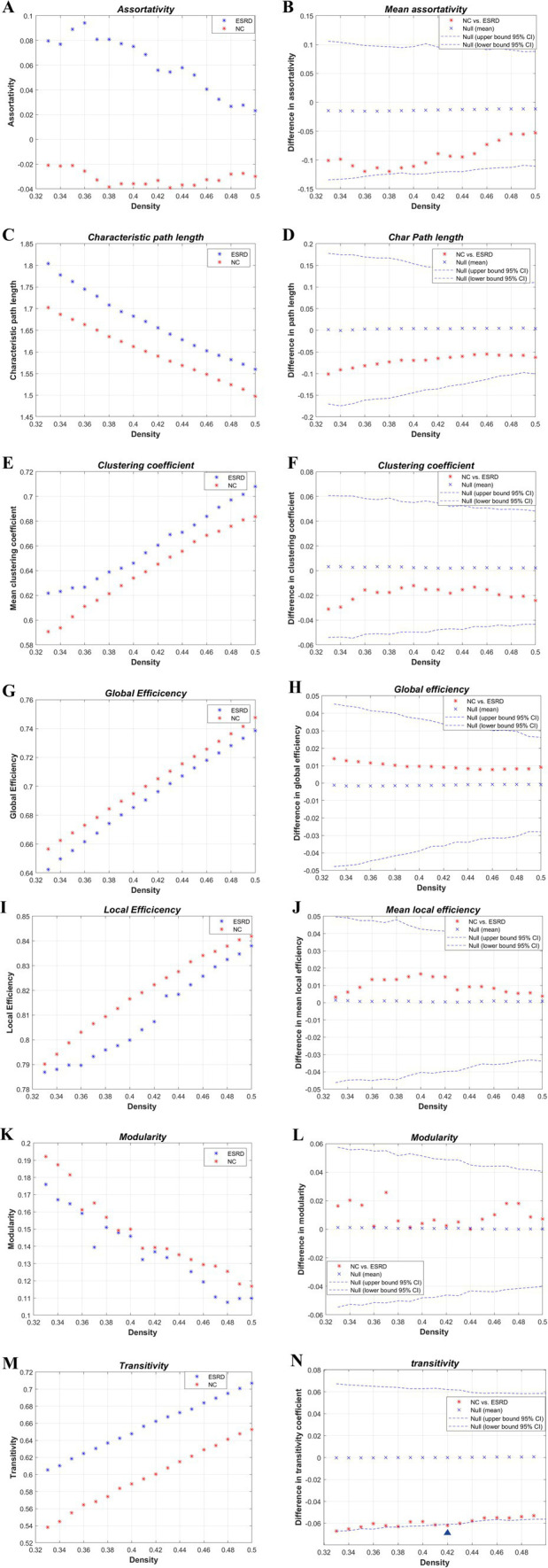
Intergroup differences in global network metrics of the cortical volume SCN between the NC and ESRD groups. [Assortativity **(A,B)**; *L*_p_ **(C,D)**; *C*_p_ **(E,F)**; global efficiency (*E*_glob_) **(G,H)**; local efficiency (*M*_LocEff_) **(I,J)**; modularity **(K,L)**; transitivity **(M,N)**. Blue triangle indicates statistically significant differences in network metrics between the two groups at a particular density].

The global network metrics (Figure 4) were further compared within a defined range of densities (0.33–0.5, 0.01 intervals), including the *C*_p_, *L*_p_, global efficiency, local efficiency, assortativity, transitivity, and modularity. Only at *D* = 0.42, was the transitivity significantly higher in the ESRD group compared to the NC group. No other indicators showed significant differences between the two groups. However, compared to the NC group, the *C*_p_, *L*_p_, and assortativity increased in the ESRD group, while global efficiency, local efficiency, and modularity decreased across most densities.

AUC analysis of the global network metrics indicated that the transitivity of the ESRD group was significantly higher than that of the NC group (*p* = 0.036). The differences in the remaining metrics between the two groups were not statistically significant (*p* > 0.05).

### 3.4 Differences between the regional networks

**Figure 5:**
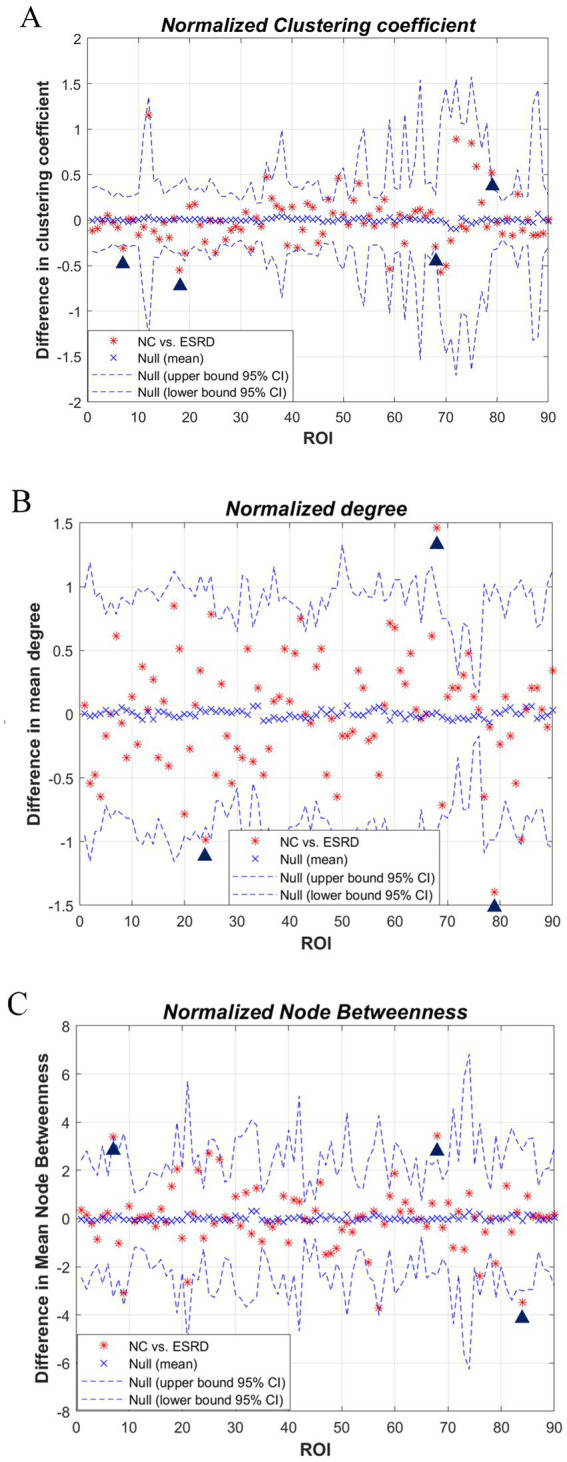
Differences in regional network metrics between the NC and ESRD groups. [**(A)** Normalized *C*_p_; **(B)** node degree; **(C)** node betweenness centrality; in plots A–C, the horizontal axes represent the corresponding ordinal numbers of brain cortical ROIs as delineated by the AAL atlas (Supplementary Table S1). The blue triangle signifies a significant difference in regional network metrics between the two groups].

We studied each index of the regional network between the two groups, i.e., the node network attribute indexes, including local clustering coefficient, degree, and node betwenness, and performed AUC analysis on them separately. The standardised local clustering coefficient of the ESRD group was obtained to be significantly lower than that of the NC group in the left Heschl gyrus, and significantly higher than that of the NC group in the left middle frontal gyrus, right rolandic operculum, and right precuneus. The degree of the ESRD group was significantly smaller than that of the NC group in the right precuneus, and significantly greater than that of the NC group in the right superior frontal gyrus, medial, and left Heschl gyrus. The node betweenness of the ESRD group was significantly smaller than that of the NC group in the left middle frontal gyrus, right precuneus, and significantly greater than that of the NC group in the right temporal pole (Figure 5, *p* < 0.05).

### 3.5 Network hubs

**Figure 6:**
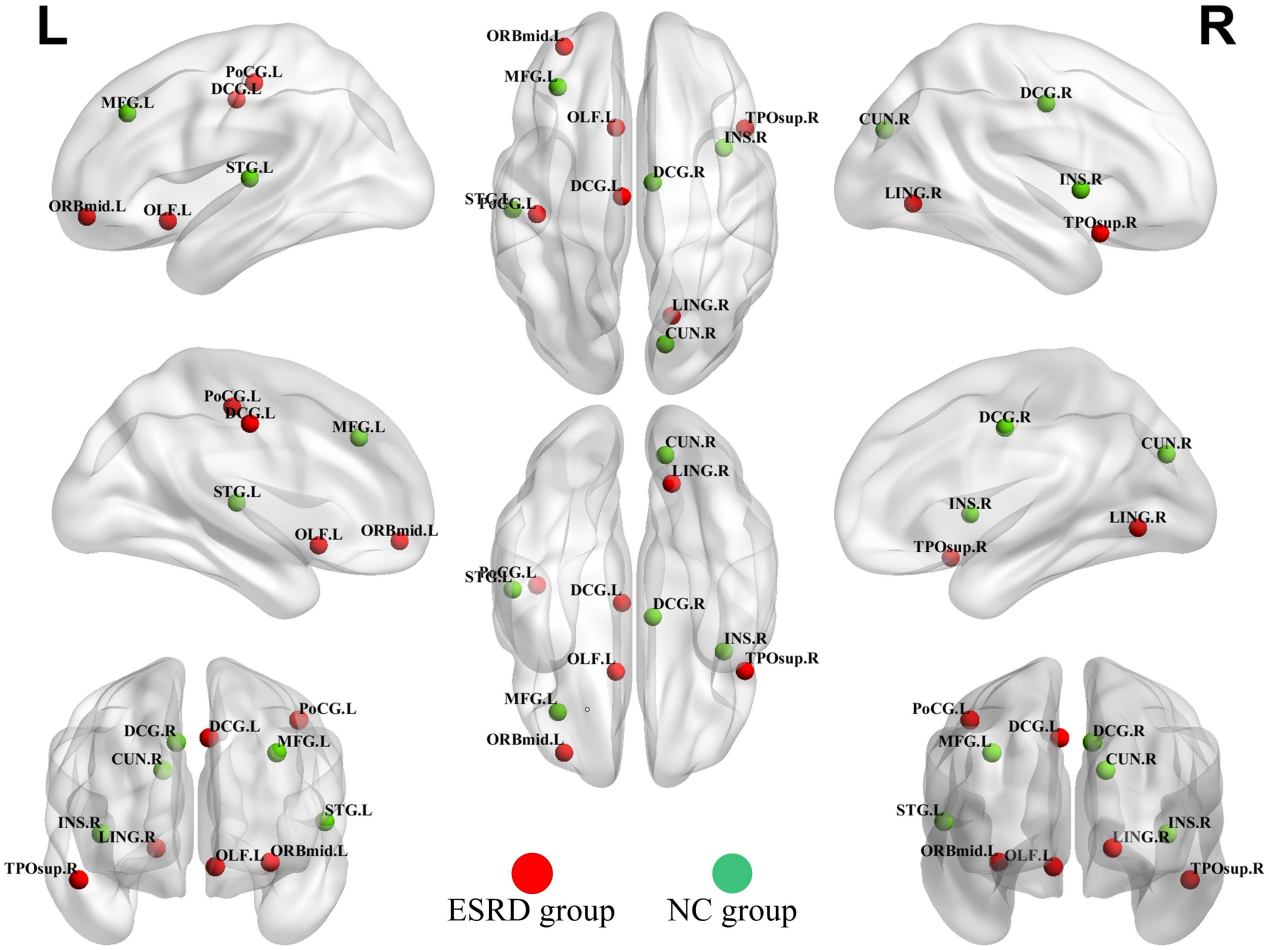
Hubs of the ESRD group and the NC group cortical volume SCN. (Green dots indicate hubs in the NC group and red dots indicate hubs in the ESRD group). Full names of the corresponding brain regions are provided in Supplementary Table S1.

Hubs were quantified by the AUC values of node betweenness centrality, with hubs defined as regions where the node betweenness centrality was greater than two times the standard deviation above the network’s average node betweenness centrality. The hubs in the ESRD group included the left middle frontal gyrus, orbital part, the left olfactory cortex, the left median cingulate and paracingulate gyri, the right lingual gyrus, the left postcentral gyrus, and the right temporal pole: superior temporal gyrus. In the NC group, the hubs included the left middle frontal gyrus, the right insula, the right median cingulate and paracingulate gyri, the right precuneus, and the left superior temporal gyrus (Figure 6).

### 3.6 Network robustness results

**Figure 7:**
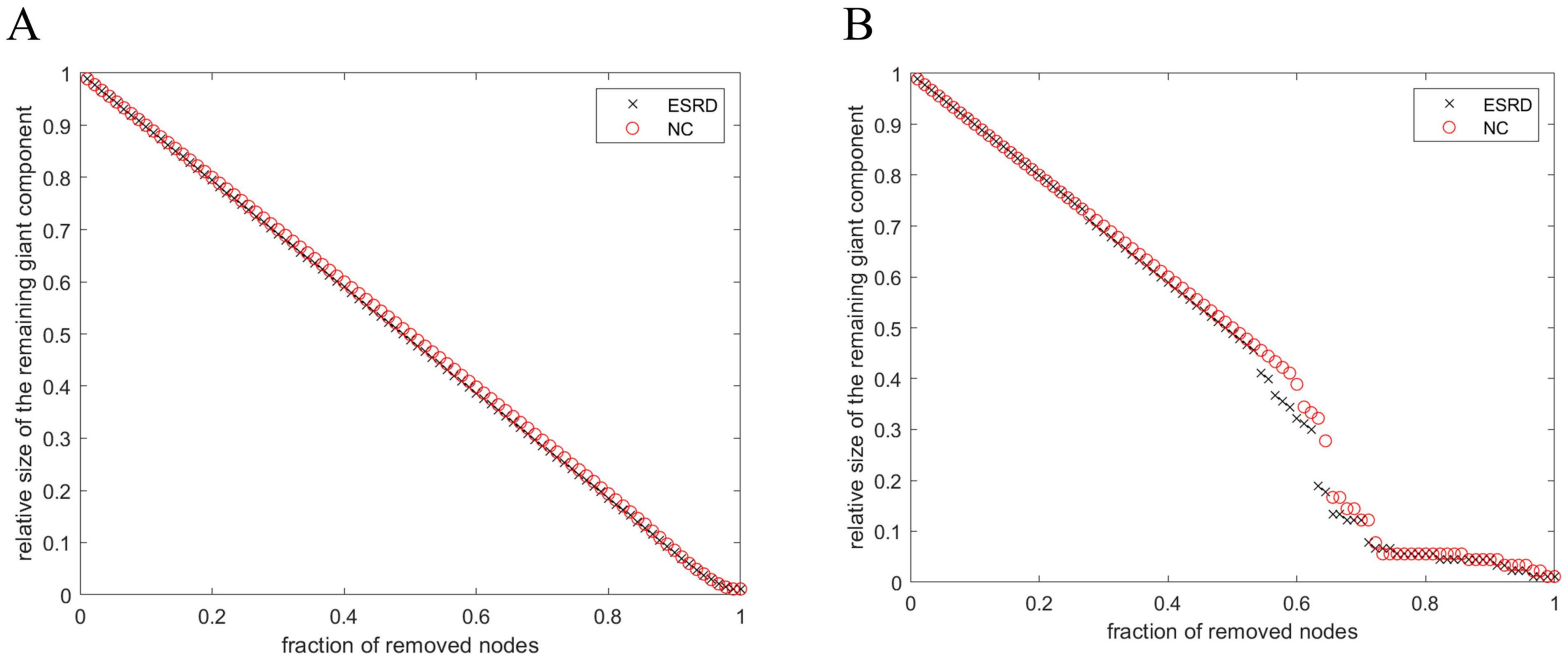
Stability of SCNs in ESRD group and NC group under random attack **(A)** and targeted attack **(B)**.

Both populations achieved network resilience in response to random and targeted attacks (Figure 7), and the network resilience in response to targeted attacks showed some degree of diminution at certain network sparsities.

## 4 Discussion

The study explored the altered structure of cortical volume SCNs in Non-CI ESRD patients by analysing sMRI data from a specific sample of ESRD patients. The study found a series of alterations in the topological properties of SCNs in Non-CI ESRD patients. Currently, no studies have demonstrated the presence of clearly targeted brain regions of injury in patients with renal failure, and relatively few macroscopic cerebral cortical structural network alterations have been explored in patients with renal failure. To progress in exploring macroscopic network relationships between neuroanatomical regions in patients with renal failure, we performed SCNs analysis of cortical volumes.

### 4.1 Analysis of cortical volume

The VBM-based study findings indicate that, compared to the normal control group, the cortical volume reduction in ESRD patients involves various brain regions, including the central executive network (CEN), the limbic association system, the salience network (SN), and the dorsal attention network (DAN). These regions exert influential roles in functions such as sensation, emotional regulation, and memory (Yeo et al., 2011; Androulakis et al., 2018).

### 4.2 Analysis of global networks

Brain networks are designed for effective information transfer and processing by striking a balance between separation and integration. Graph theoretic analysis can quantitatively analyse the separation and integration of brain networks (Rubinov and Sporns, 2010). In this paper, we found that although the SCN of Non-CI ESRD patients still retains the small-world topology property, the characteristic path lengths and clustering coefficients increase in most density distributions, and the network characteristics of Non-CI ESRD patients are gradually converging to regular networks (Latora and Marchiori, 2001). Similar alterations in the topological properties of rules are also seen in brain networks of people with AD or schizophrenia disorders (Bullmore and Sporns, 2009). This shift indicates that the structural covariance network in Non-CI ESRD patients shows reduced segregation between various brain regions and compromised segregation function. This can result in outcomes like lower efficiency in regional information processing, higher cognitive load, and diminished neural network plasticity. It suggests that brain regions that previously processed information independently may now interfere with each other, and the brain requires extra resources to differentiate and process information from different regions effectively.

Transitivity is a metric used to quantify network segregation; therefore, greater transitivity indicates that ROIs tend to form highly interconnected clusters. The presence of significantly reduced transitivity has been found in studies of brain network alterations in people with systemic lupus erythematosus (Preziosa et al., 2020), cortical dysplasia (Lee et al., 2021), and those at risk for high-risk psychiatric disorders (Prasad et al., 2023). In a study of the effect of structural connectivity on cognitive performance in multiple sclerosis, Lopez-Soley et al. found that measures of transitivity of brain structural networks were positively correlated with cognitive scores (Lopez-Soley et al., 2020), suggesting that patients with multiple sclerosis with increased transitivity have better cognitive performance, and thus suggesting that increased transitivity is an enhancement of the efficacy of the whole-brain network, and that increased transitivity may act as a structural reorganisation of the brain in ESRD patients. The increased transitivity may act as a compensatory mechanism for the reorganisation of brain structures in ESRD patients to ensure that the transmission efficiency of the whole-brain network is not significantly altered.

In conclusion, this study demonstrates that the SCNs pattern of overall grey matter volume in ESRD patients is shifting from a small-world to a regular network. Certain genetic and immunological factors, such as apolipoprotein E (APOE), a complex protein crucial for neuronal repair and plasticity following injury (Samatovicz, 2000), may significantly influence brain structural network alterations. A study by Bijkerk et al. (2022) has confirmed that circulating angiopoietin-2 and decreased levels of specific microRNAs (particularly miR-132) may serve as important biomarkers in elderly ESRD patients. They are correlated with quantitative changes in white matter hyperintensities (WMH) volume and cognitive decline in the brain. These factors could lead to changes in the connectivity of the SCNs in ESRD patients, causing a reorganization of the network structure.

### 4.3 Analysis of regional networks

Analysis of node betweenness and degree measures can assess differences in brain centrality interactions between two groups. This study found significant decreases of RE in the right precuneus and the left middle frontal gyrus of the ESRD group, wherein the right precuneus showed a more pronounced decrease, resulting in a severe network loss and a significant reduction in network bridges. Many neurofunctional connectivity studies have also reported that ESRD patients have significantly reduced node mediation in the right precuneus and middle frontal gyrus, and that these regions may also be associated with neurovascular coupled NVC dysfunction, which correlates with cognitive impairment in ESRD patients (Yue et al., 2021; Hu et al., 2024). In in patients with Non-CI ESRD, the precuneus serves multiple roles, including involvement in the core of the DMN and its situational memory network as well as the paracentral gyrus network, which are critical for complex cognitive functions (Dadario and Sughrue, 2023).

The middle frontal gyrus, a highly interconnected cortical area implicated in attentional processing, working memory, and language generation and comprehension, plays a key role in the frontoparietal network (FPN) (Briggs et al., 2021). In conclusion, our study offers a macroscopic perspective on the markedly reduced covariation network capacity in the right precuneus and the left middle frontal gyrus of ESRD patients, providing a basis for the earlier detection of cognitive changes and macroscopic alterations in brain structure and network.

Non-CI ESRD patients also exhibit regions of significantly increased RE, such as the right superior medial frontal gyrus, left transverse temporal gyrus, and right temporal pole. The mechanisms behind these high centrality regions remain unclear but may be associated with protective factors identified in existing research. Aerobic exercise during dialysis sessions has been shown to reduce the incidence of cardiovascular diseases in ESRD patients, and high-volume hemodiafiltration enhances hemodynamic stability and better preserves cerebral perfusion, potentially mitigating the accelerated cognitive decline and progression of white matter lesions in ESRD patients (Isnard-Rouchon and Coutard, 2017; Kim, 2018). Further research into the relationship between brain structure or functional connectivity and clinical indicators in ESRD could elucidate the contributing causes. The current findings also indicate that potential secondary neuroendocrine factors may impact the connectivity of large-scale brain structural networks (Hosseini et al., 2012).

### 4.4 Analysis of network hubs

In the NC group, hubs were predominantly partly overlapped with the hubs observed in the brain networks of healthy adults (Ni et al., 2014).

The presence of brain reorganizational plasticity predisposes the right temporal pole and right superior temporal gyrus to serve as focal points for altered compensatory networks in ESRD patients. A recent diffusion tensor imaging DTI investigation into ESRD patients revealed a reduction in average white matter fiber tracts across the board. Notably, however, there was a significant increase in white matter fiber tracts within the bilateral superior temporal gyri (Ma et al., 2022), indicating a compensatory mechanism for white matter fibers in these regions among ESRD patients. Previous research on the diffusion tensor imaging DTI network in long-term hemodialysis patients with ESRD revealed nodes exhibiting elevated clustering coefficients (Chou et al., 2019). This increased connectivity among local nodes and enhanced efficiency of information transfer imply the existence of compensatory mechanisms in ESRD patients. This phenomenon was further supported by the analysis of functional brain networks in ESRD patients via fMRI, which identified areas of enhanced connectivity within the DMN and the SN. These findings suggest that specific connectivity changes and neuropathological alterations may be linked to cognitive compensation in ESRD patients (Chang et al., 2021; Huang et al., 2018; Wu et al., 2020).

### 4.5 Network robustness analysis

The dynamic behaviour of a network may be closely related to its underlying topology (Kaiser and Hilgetag, 2004). Recent studies have indicated that a modular architecture is associated with the maintenance of the brain’s robustness (Chen et al., 2021). Thus, variations in network parameters reflect disruptions to the network’s general performance. This study demonstrates that compared to the control group, brain networks of Non-CI ESRD patients showed a more pronounced decrease in topological stability in the face of target attacks, which may be related to histopathological changes in the brain cortex of Non-CI ESRD patients.

### 4.6 Limitations and prospects

This study is accompanied by certain limitations. Initially, it was a cross-sectional design, precluding the assessment of the direct dissociated effects of ESRD on network measures. Future research should consider longitudinal assessments of network metrics in patients with ESRD to mitigate this limitation. Furthermore the brain structural network changes observed in ESRD patients at the individual level merit further investigation. Concurrently, correlation analyses should be performed in tandem with pertinent clinical markers to delve into the impact of these markers on brain network alterations.

## Data Availability

The raw data supporting the conclusions of this article will be made available by the authors, without undue reservation.

## Footnotes

^1^ https://www.mathworks.com/products/matlab.html
