# Supplementary material for: Cortical structural network characteristics in non-cognitive impairment end-stage renal disease
Source: Front Neurosci. 2024 Nov 13;18:1467791. doi: 10.3389/fnins.2024.1467791 (PMC11599166; doi:10.3389/fnins.2024.1467791)
Supplement: Supplementary file 1 [file Table_1.DOCX]

**Supplementary Table 1 Anatomical Automatic Labeling(AAL) Area Comparison Table**

| **serial number** | **abbreviation 1** | **abbreviation 2** | **Full name** |
| --- | --- | --- | --- |
| 1 | Precentral_L | PreCG.L | Precental gyrus |
| 2 | Precentral_R | PreCG.R | Precental gyrus |
| 3 | Frontal_Sup_L | SFGdor.L | Superior frontal gyrus, dorsolateral |
| 4 | Frontal_Sup_R | SFGdor.R | Superior frontal gyrus, dorsolateral |
| 5 | Frontal_Sup_Orb_L | ORBsup.L | Superior frontal gyrus, orbital part |
| 6 | Frontal_Sup_Orb_R | ORBsup.R | Superior frontal gyrus, orbital part |
| 7 | Frontal_Mid_L | MFG.L | Middle frontal gyrus |
| 8 | Frontal_Mid_R | MFG.R | Middle frontal gyrus |
| 9 | Frontal_Mid_Orb_L | ORBmid.L | Middle frontal gyrus, orbital part |
| 10 | Frontal_Mid_Orb_R | ORBmid.R | Middle frontal gyrus, orbital part |
| 11 | Frontal_Inf_Oper_L | IFGoperc.L | Inferior frontal gyrus, opercular part |
| 12 | Frontal_Inf_Oper_R | IFGoperc.R | Inferior frontal gyrus, opercular part |
| 13 | Frontal_Inf_Tri_L | IFGtriang.L | Inferior frontal gyrus, triangular part |
| 14 | Frontal_Inf_Tri_R | IFGtriang.R | Inferior frontal gyrus, triangular part |
| 15 | Frontal_Inf_Orb_L | ORBinf.L | Inferior frontal gyrus, orbital part |
| 16 | Frontal_Inf_Orb_R | ORBinf.R | Inferior frontal gyrus, orbital part |
| 17 | Rolandic_Oper_L | ROL.L | Rolandic operculum |
| 18 | Rolandic_Oper_R | ROL.R | Rolandic operculum |
| 19 | Supp_Motor_Area_L | SMA.L | Supplementary motor area |
| 20 | Supp_Motor_Area_R | SMA.R | Supplementary motor area |
| 21 | Olfactory_L | OLF.L | Olfactory cortex |
| 22 | Olfactory_R | OLF.R | Olfactory cortex |
| 23 | Frontal_Sup_Medial_L | SFGmed.L | Superior frontal gyrus, medial |
| 24 | Frontal_Sup_Medial_R | SFGmed.R | Superior frontal gyrus, medial |
| 25 | Frontal_Mid_Orb_L | ORBsupmed.L | Superior frontal gyrus, medial orbital |
| 26 | Frontal_Mid_Orb_R | ORBsupmed.R | Superior frontal gyrus, medial orbital |
| 27 | Rectus_L | REC.L | Gyrus rectus |
| 28 | Rectus_R | REC.R | Gyrus rectus |
| 29 | Insula_L | INS.L | Insula |
| 30 | Insula_R | INS.R | Insula |
| 31 | Cingulum_Ant_L | ACG.L | Anterior cingulate and paracingulate gyri |
| 32 | Cingulum_Ant_R | ACG.R | Anterior cingulate and paracingulate gyri |
| 33 | Cingulum_Mid_L | DCG.L | Median cingulate and paracingulate gyri |
| 34 | Cingulum_Mid_R | DCG.R | Median cingulate and paracingulate gyri |
| 35 | Cingulum_Post_L | PCG.L | Posterior cingulate gyrus |
| 36 | Cingulum_Post_R | PCG.R | Posterior cingulate gyrus |
| 37 | Hippocampus_L | HIP.L | Hippocampus |
| 38 | Hippocampus_R | HIP.R | Hippocampus |
| 39 | ParaHippocampal_L | PHG.L | Parahippocampal gyrus |
| 40 | ParaHippocampal_R | PHG.R | Parahippocampal gyrus |
| 41 | Amygdala_L | AMYG.L | Amygdala |
| 42 | Amygdala_R | AMYG.R | Amygdala |
| 43 | Calcarine_L | CAL.L | Calcarine fissure and surrounding cortex |
| 44 | Calcarine_R | CAL.R | Calcarine fissure and surrounding cortex |
| 45 | Cuneus_L | CUN.L | Cuneus |
| 46 | Cuneus_R | CUN.R | Cuneus |
| 47 | Lingual_L | LING.L | Lingual gyrus |
| 48 | Lingual_R | LING.R | Lingual gyrus |
| 49 | Occipital_Sup_L | SOG.L | Superior occipital gyrus |
| 50 | Occipital_Sup_R | SOG.R | Superior occipital gyrus |
| 51 | Occipital_Mid_L | MOG.L | Middle occipital gyrus |
| 52 | Occipital_Mid_R | MOG.R | Middle occipital gyrus |
| 53 | Occipital_Inf_L | IOG.L | Inferior occipital gyrus |
| 54 | Occipital_Inf_R | IOG.R | Inferior occipital gyrus |
| 55 | Fusiform_L | FFG.L | Fusiform gyrus |
| 56 | Fusiform_R | FFG.R | Fusiform gyrus |
| 57 | Postcentral_L | PoCG.L | Postcentral gyrus |
| 58 | Postcentral_R | PoCG.R | Postcentral gyrus |
| 59 | Parietal_Sup_L | SPG.L | Superior parietal gyrus |
| 60 | Parietal_Sup_R | SPG.R | Superior parietal gyrus |
| 61 | Parietal_Inf_L | IPL.L | Inferior parietal, but supramarginal and angular gyri |
| 62 | Parietal_Inf_R | IPL.R | Inferior parietal, but supramarginal and angular gyri |
| 63 | SupraMarginal_L | SMG.L | Supramarginal gyrus |
| 64 | SupraMarginal_R | SMG.R | Supramarginal gyrus |
| 65 | Angular_L | ANG.L | Angular gyrus |
| 66 | Angular_R | ANG.R | Angular gyrus |
| 67 | Precuneus_L | PCUN.L | Precuneus |
| 68 | Precuneus_R | PCUN.R | Precuneus |
| 69 | Paracentral_Lobule_L | PCL.L | Paracentral lobule |
| 70 | Paracentral_Lobule_R | PCL.R | Paracentral lobule |
| 71 | Caudate_L | CAU.L | Caudate nucleus |
| 72 | Caudate_R | CAU.R | Caudate nucleus |
| 73 | Putamen_L | PUT.L | Lenticular nucleus, putamen |
| 74 | Putamen_R | PUT.R | Lenticular nucleus, putamen |
| 75 | Pallidum_L | PAL.L | Lenticular nucleus, pallidum |
| 76 | Pallidum_R | PAL.R | Lenticular nucleus, pallidum |
| 77 | Thalamus_L | THA.L | Thalamus |
| 78 | Thalamus_R | THA.R | Thalamus |
| 79 | Heschl_L | HES.L | Heschl gyrus |
| 80 | Heschl_R | HES.R | Heschl gyrus |
| 81 | Temporal_Sup_L | STG.L | Superior temporal gyrus |
| 82 | Temporal_Sup_R | STG.R | Superior temporal gyrus |
| 83 | Temporal_Pole_Sup_L | TPOsup.L | Temporal pole: superior temporal gyrus |
| 84 | Temporal_Pole_Sup_R | TPOsup.R | Temporal pole: superior temporal gyrus |
| 85 | Temporal_Mid_L | MTG.L | Middle temporal gyrus |
| 86 | Temporal_Mid_R | MTG.R | Middle temporal gyrus |
| 87 | Temporal_Pole_Mid_L | TPOmid.L | Temporal pole: middle temporal gyrus |
| 88 | Temporal_Pole_Mid_R | TPOmid.R | Temporal pole: middle temporal gyrus |
| 89 | Temporal_Inf_L | ITG.L | Inferior temporal gyrus |
| 90 | Temporal_Inf_R | ITG.R | Inferior temporal gyrus |
